# Titrating the Translational Relevance of a Low-Level Repetitive Head Impact Model

**DOI:** 10.3389/fneur.2022.857654

**Published:** 2022-06-16

**Authors:** Masen L. Boucher, Grace Conley, Jordan Nowlin, Jianhua Qiu, Keisuke Kawata, Jeffrey J. Bazarian, William P. Meehan, Rebekah Mannix

**Affiliations:** ^1^Harvard Medical School, Division of Emergency Medicine, Boston Children's Hospital, Boston, MA, United States; ^2^Department of Pediatrics, Harvard Medical School, Boston, MA, United States; ^3^Department of Kinesiology, Indiana University, Bloomington, IN, United States; ^4^Department of Emergency Medicine, University of Rochester School of Medicine and Dentistry, Rochester, NY, United States; ^5^Harvard Medical School, Division of Sports Medicine, Boston Children's Hospital, Boston, MA, United States; ^6^The Micheli Center for Sports Injury Prevention, Waltham, MA, United States

**Keywords:** repetitive head impact, RHI, sub-concussion, neuroinflammation, blood-brain biomarkers, traumatic brain injury, TBI

## Abstract

Recently, there has been increased attention in the scientific community to the phenomenon of sub-concussive impacts, those hits to the head that do not cause the signs and symptoms of a concussion. Some authors suggest that sub-concussive impacts may alter behavior and cognition, if sustained repetitively, but the mechanisms underlying these changes are not well-defined. Here, we adapt our well-established weight drop model of repetitive mild traumatic brain injury (rmTBI) to attempt to produce a model of low-level repetitive head impacts (RHI). The model was modified to eliminate differences in latency to right following impact and gross behavioral changes after a single cluster of hits. Further, we varied our model in terms of repetition of impact over a 4-h span to mimic the repeated sub-concussive impacts that may be experienced by an athlete within a single day of play. To understand the effects of a single cluster of RHIs, as well as the effect of an increased impact frequency within the cluster, we evaluated classical behavioral measures, serum biomarkers, cortical protein quantification, and immunohistochemistry both acutely and sub-acutely following the impacts. In the absence of gross behavioral changes, the impact protocol did generate pathology, in a dose-dependent fashion, in the brain. Evaluation of serum biomarkers revealed limited changes in GFAP and NF-L, which suggests that their diagnostic utility may not emerge until the exposure to low-level head impacts reaches a certain threshold. Robust decreases in both IL-1β and IL-6 were observed in the serum and the cortex, indicating downregulation of inflammatory pathways. These experiments yield initial data on pathology and biomarkers in a mouse model of low-level RHIs, with relevance to sports settings, providing a starting point for further exploration of the potential role of anti-inflammatory processes in low-level RHI outcomes, and how these markers may evolve with repeated exposure.

## Introduction

Recently, there has been increased concern over the consequences of repetitive head impacts (RHIs) on long-term brain health. The term “RHIs” is used to capture the effects of recurrent head impacts, including those head impacts that do not result in overt symptomatology (i.e. sub-concussive head impacts) (1–5). Particular concern has been directed to sub-concussive RHI exposures, where the transfer of mechanical energy can result in altered axonal integrity, without an easily detectable clinical signal to guide decision making around limiting these exposures. Indeed, some experts have suggested that it is the accumulation of sub-concussive RHIs, rather than concussions, that is responsible for the neuropathological syndrome of chronic traumatic encephalopathy (CTE) (6). As a result, there has been growing interest in the scientific field to monitor RHIs in real-time, theoretically, to prevent the accumulation of a certain threshold of RHI exposure.

Several studies have suggested that blood-brain biomarkers might be useful to monitor molecular changes in the brain after RHI exposure. Neselius et al. found increases in plasma tau within a week of a sparring session in amateur boxers (7). Investigation into biomarker changes in collegiate football players over the course of one practice revealed increases in neurofilament light-chain (NF-L, marker of axonal integrity) that were moderated by the magnitude and frequency of head impact, and associated with the linear and rotational acceleration sustained (8). The use of a controlled soccer ball heading model (9) led to increased NF-L levels 24 h after 10 headers in 10-min (9), as well as 40 headers in 20-min (10), with the latter leading to persistently elevated levels up to 22 days after the session, and notably, subtle symptomatology as well (elevated symptoms score on The Standardized Assessment of Concussion−3rd edition [SCAT] 3). A few studies have correlated changes in blood-brain biomarkers with changes in imaging biomarkers (11–13), further supporting the link between RHIs and changes in the brain microstructure. Direct tissue correlates of these changes, however, are not possible in the clinical setting.

Preclinical studies are needed to determine tissue correlates of blood-brain biomarkers after RHI exposure and to investigate further questions about dose-response. To date, the rodent literature has varied in terms of the number of impacts (single vs. repetitive), impact interval (a daily single impact over multiple days vs. multiple impacts each day over multiple days), and impact magnitude (14–20). While the initial work has attempted to address the phenomenon of sub-concussion, much of it has fallen short in how accurately it models the repetitive impacts experienced by humans. In particular, impact frequency and interval should take into account the practice and play schedules of the athletes at risk. Preclinical studies using longer intervals between RHI clusters, such as 3 days (14), or even 1 week (17), are unrepresentative of the time between contact practices and/or games in sports such as soccer and American football (21). While preclinical RHI administrations with a 24-h interval are a better representation of sport-related exposure, inclusion of just one impact within each session misses the repetitiveness or “clustering” of the impacts sustained during a single contact practice or game, which can be as high as 41 in collegiate football players (8, 15). As rodent models of RHI allow for the opportunity to evaluate dose response in a systematic manner, faithful and intentional manipulation of the impact interval is key to furthering our understanding of these factors. Similar to issues of the number of hits and interval, titrating the impact magnitude to replicate a sub-concussive hit, avoiding conflation with mTBI (or concussion), is important. Clinical work examining the hits sustained over the course of collegiate football and ice hockey (men and women) seasons demonstrates that on average, players sustain 469 hits across this time, yet there were only post-season deficits on the California Verbal Learning Test (CVLT) (22). Similarly, Gysland et al. determined collegiate football players sustained an average of 1,177 impacts over the course of a season and found limited changes on measures traditionally used to evaluate neurologic impairment, with a modest increase in symptoms on the self-report Graded Symptom Checklist (GSC) (23). When such an immense RHI load over a several month season yields only subtle changes, it suggests that a rodent RHI paradigm that yields robust behavioral changes following three-to-four hits over a several week period (14, 17) likely represents a more severe impact, more similar to concussion than to sub-concussive blows. While the two forms of head trauma likely have commonalities, there is no certainty that the underlying mechanisms of pathology are comparable between the two.

Another important limitation of rodent models intended to represent sub-concussive head impacts is the inability to investigate both the signs and symptoms of TBI—rodent behavioral tests allow confirmation of signs resulting from TBI, but the associated symptoms, such as headache, sensitivity to sound, or nausea, cannot be examined in rodents. In addition, many of the studies to date have used a limited set of behavioral tests, raising the possibility that the models result in unmeasured changes in functional outcomes. Here, as with other rodent work, we rely on the absence of signs of concussion using a robust set of behavioral assays, but cannot confidently comment on the absence of symptoms. Thus, the term “low-level” is used throughout the remainder of this study, as opposed to sub-concussive, recognizing that the animals may have suffered concussions as currently defined clinically. Nonetheless, attempting to model sub-concussive RHI is important given the increasing scientific concern that repeated sub-concussive head impacts over an extended period may cumulatively induce significant structural and functional changes to the brain. Despite this growing worry, no pre-clinical study of which we are aware has addressed both the dose response to low-level RHIs and the temporal expression of changes in blood-brain biomarkers and tissue pathology following low-level RHIs. These data are needed to provide a mechanistic basis which can inform research into how RHI exposures may be additive over time, and the potential threshold at which RHIs become detrimental.

In light of these gaps in the literature, we aim to investigate the effects of a single cluster of low-level RHIs on biomarkers, brain histology, and behavior from acute to subacute time points, in an attempt to better understand the connection between real-time biomarkers and changes in the brain after low-level head impacts. To imitate the clustering of low-level RHIs experienced by an athlete within a day of play, we have modified a weight drop model to deliver a less severe, closed-head impact without eliciting signs of concussion, as well as varied our paradigm in terms of the repetition of the impacts and the interval between impacts, within a single session. We hypothesize that low-level impacts will, in a dose-dependent manner, lead to subtle changes in behavior and cytokine levels in both the brain and blood, with a relationship between a subset of biomarkers and neural and behavioral outcomes, but that the low-level impacts will have limited effects on gross histopathology.

## Materials and Methods

All experiments were approved by the Boston Children's Hospital Institutional Animal Care And Use Committee and complied with the NIH Guide for the Care and Use of Laboratory Animals. Eighty (age 8 weeks) male C57BL/6 mice were obtained from the Jackson Laboratories (Bar Harbor, ME) for these experiments and housed in a pathogen free environment with inverse 12-h day-night cycles. Food and water were provided ad libitum to all animals.

### Impact Paradigm

**Figure 1:**
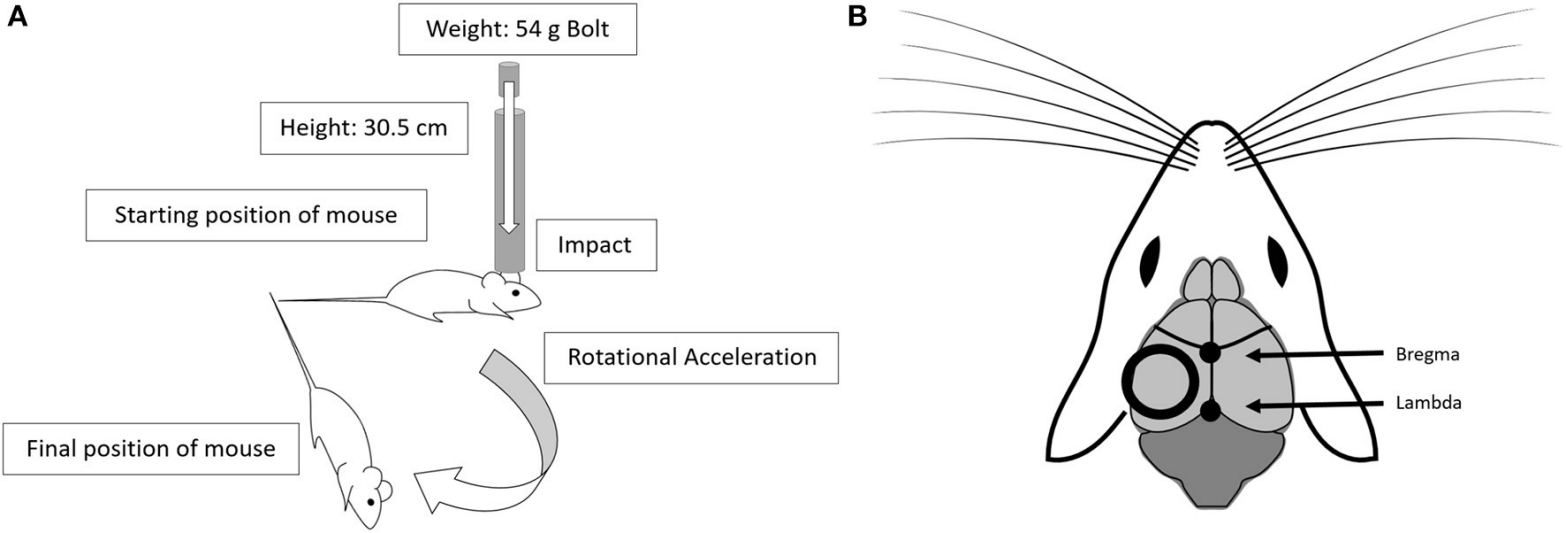
The low-level head impact utilizes the drop weight method. **(A)** This model uses a 54 g bolt dropped from 30.5 cm, through a guide tube, leading to impact and rotational acceleration. **(B)** The position of the impact alternates between left and right side impacts, and is centered over the dorsal surface of the head, positioned between the coronal and lamboid structures. Diagram adapted from open source drawing (28).

Mice were randomized to either a low-level repetitive hit paradigm (repetitive head impact, RHI) with varied hit frequencies, or a sham procedure. As outlined in the introduction, due to methodological limitations related to rodent models, we recognize that we cannot confirm whether this impact paradigm represents either a sub-concussive hit or a very mild concussion. This paradigm was similar to our repetitive mild TBI (rmTBI) (106.7 cm drop height), which has been previously described (24, 25), with several important modifications. All mice were anesthetized for 45 s using 4% isoflurane in oxygen. Anesthetic depth was confirmed via lack of response to a toe pinch. Anesthetized mice receiving an impact were placed on a delicate tissue (Kimwipe, Irving, TX) in a prone position and secured by the tail. The animal was positioned directly under a hollow iron guide tube and a 54-gram metal bolt was dropped through the tube to impact the dorsal surface of the head right above the right or left ear between the coronal and lamboid sutures (Figure 1). In each mouse, the repetitive hits were alternatively delivered to each side of the head, such that the first, third, fifth, and seventh hits were always delivered to the right side, while the second, fourth, sixth, and eighth hits were delivered to the left side. Our decision to offset the impact to the side of the head was influenced by the recent discovery of visual dysfunction following our center hit rmTBI model (26). As evidenced by the Morris water maze visible trial results presented here, there is no difference in functional vision between sham animals and animals who received the low-level hits to the side of the head. While the impact is no longer centralized, as we have demonstrated previously, the weight drop protocol generates a global injury without focal damage at the site of impact (24, 25), so there is no reason to suspect that a non-centered impact would only affect one hemisphere of the brain. Additionally, we chose a protocol that delivers an even number of impacts to both sides of the head to ensure global effects. The 54-gram bolt was released from a height of 30.5 cm, thus delivering 0.16 joules to the head, and resulting in rotational acceleration through the Kimwipe. From removal from isoflurane to completion of impact, the procedure took approximately 15 s. After the impact, the mouse was placed in a left lateral lying position to regain consciousness in room air. This impact procedure, at both the 106.7 cm and 30.5 cm drop heights, does not result in hemorrhage, hematoma, or cranial fracture (24, 25, 27). Mice in the low-level RHI group either underwent 2 hits in 4 h (*n* = 20, 2 hits/4 h; 2-h interval between hits), 4 hits in 4 h (*n* = 20, 4 hits/4 h; 1-h interval between hits), or 8 hits in 4 h (*n* = 20, 8 hits/4 h; 30-min interval between hits). A separate group underwent a sham procedure that consisted of anesthesia exposure at the same frequencies and intervals as the low-level RHI group (sham 2/4 h: *n* = 6, sham 4/4 h: *n* = 7, sham 8/4 h: n= 7). The latency to right was recorded for all groups and was defined as the time from which the mouse was removed from anesthesia to the time in which it was sternal, or had “righted” itself.

### Timeline

**Figure 2:**
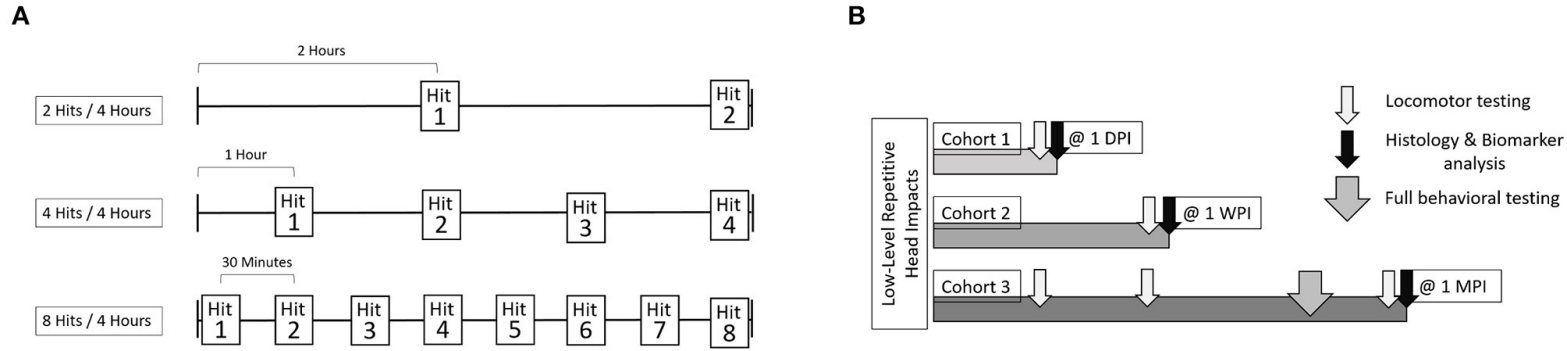
The experimental outline includes three cohorts of animals. Each cohort was comprised of 4 groups, sham, 2 hits in 4 h, 4 hits in 4 h, and 8 hits in 4 h. **(A)** For each of these groups, both the number of impacts sustained, as well as interval between impacts, differed. For the 2 hits/4 h group, there was a 2-h interval between impacts. For the 4 hits/4 h group, there was a 1-h interval between impacts. For the 8 hits/4 h group, there was a 30-min interval between impacts. Shams were split across each of these groups, so that some sham animals received isoflurane exposures alongside the 2 hit group, others alongside the 4 hits group or the 8 hits group. **(B)** Cohort 1 received locomotor testing at 1-day post-impact and were sacrificed for histological outcomes immediately following. Cohort 2 received locomotor testing at 1 week and were sacrificed for histological outcomes at that time as well. Cohort 3 received locomotor testing at 1-day post-impact, 1-week post-impact, and 1-month post-impact. Cohort 3 also underwent “full behavioral testing” starting at 2-weeks post-impact including Rotarod, Open Field Test, Elevated Plus Maze, Morris Water Maze, and the Von Frey test. Cohort 3 was sacrificed for histological outcomes at 1-month post-impact.

From each RHI group, as well as each sham group, animals were further divided into cohorts based on timing of behavioral outcome and sacrifice. Cohort 1 performed the Locomotor test the day following the impacts and were sacrificed immediately after at 1-day post impact (1 DPI). Cohort 2 performed the Locomotor test 1-week-post-impact (1 WPI) and were sacrificed immediately following. Cohort 3 was tested on the Locomotor test at both 1-day and 1-week post-impact, and then began a battery of behavioral tests at 2-weeks post-impact and were sacrificed 1-month-post-impact (1 MPI). Cohorts 1 and 2 each consisted of 24 animals (*n* = 6 per impact condition including sham, per cohort). Cohort 3 included 32 animals (*n* = 8 per impact condition including sham) (Figure 2).

### Behavioral Testing

Cohort 1 and Cohort 2 performed only the Locomotor test the day after impact and a week after impact, respectively. Cohort 3 was tested on the Locomotor test at 1 day and 1 week after impact, and then began a longer set of behavioral tests 2 weeks after their impacts. The Cohort 3 mice were assessed on Rotarod, Elevated Plus Maze, Locomotor activity and Open Field, Morris Water Maze, and the Von Frey test at that time point. All behavioral testing was conducted by investigators blinded to impact status. All tests were included due to their relevance to mild TBI, as well repetitive mild TBI, with many serving as negative controls to ensure that our low-level impacts were not generating concussion-like signs, and, hence, were not too severe for what we are attempting to model.

### Locomotor Activity and Open Field

Locomotor activity was assessed using the Locomotor test (29, 30) at 1 day and 1 week after the impacts, and both locomotor activity and anxiety-like behavior were tested at 1 month using the Locomotor and Open Field test. Anxiety is known to be modulated post-TBI (31, 32), with behavioral replication within pre-clinical models (33–35). While we do not expect large affective changes, such as anxiety-like behavior, in our model, changes in locomotor behavior, which is also measured by this test, could be a subtle and more appropriate result of our paradigm. The arena consisted of a plastic transparent box (40 cm x 40 cm x 30 cm) with 32 infrared beams (16X and 16Y) crossing the X and Y axes to plot the animal's movements. The arena was virtually split into two concentric square regions; the peripheral region (outer ring of 10 cm width) and the center region (inner 20 cm x 20 cm). Each mouse was placed in the arena facing the back wall and allowed to roam freely for 10 min while movements were recorded using the MotorMonitor II tracking software (Kinder Scientific, Poway, CA). The time spent in each zone and the distance traveled were measured and used as metrics of anxiety-like behavior and motor activity. Locomotor activity was defined as the sum of the distance traveled in all zones, with longer distances constituting higher activity. More time spent in the center region indicated decreased anxiety, whereas more time spent in the peripheral region indicated increased anxiety. The arena was cleaned between each trial using Peroxigard Wipes (Virox Technologies, ON, Canada).

### Rotarod

Motor function was assessed 1 month after the last impact, using the Rotarod assay (36). Deficits in motor coordination have been identified in the clinical setting and replicated using pre-clinical models of mild TBI (27, 37, 38). Here, we include this test as a measure to ensure that our RHI intervention does not lead to gross changes in motor performance. The device, a ROTO-ROD Series 8 (IITC, Woodland Hills, CA), consists of several lanes with 3.18-cm diameter rotating drums. Rotarod testing was conducted over 3 days. For each test, the animal was placed on a rotating drum and the latency to fall (s) was recorded. Habituation/training was conducted on the first day as follows. For 5 min, each animal was placed on the drum, which was set to a rotation of 4 RPM with no acceleration. If the animal fell off, it was gently placed back on to the drum to continue until the 5-min mark was reached. Testing was conducted on days 2 and three. Animals were placed on the drum, which began rotating at 3 RPM with an acceleration of 0.1 RPM/second. Each mouse completed 4 trials/day on testing days.

### Elevated Plus Maze

Anxiety was assessed using the Elevated Plus Maze (EPM), as previously described (25, 27), at 1-month post-impact. Again, anxiety is known to be modulated post-TBI (31, 32), and EPM has faithfully been used to detect such deficits in rodent models of TBI (34, 35). The EPM apparatus (Lafayette Instrument, Lafayette, IN) is an elevated (85 cm tall) plus-shaped platform that consists of two open and two closed arms (30 x 5 cm) extending out opposite from each other, with a square-shaped center (5 x 5 cm, decision zone). The lights in the room were dimmed and the open arms were illuminated using lamps. The lamps were placed equidistant from the maze so that the end of each open arm received 30 lux. Mice were placed in the decision zone of the maze facing a closed arm at the start of each test and allowed to roam freely through the apparatus for 5 min. The Noldus Ethovision XT 11.5 (Wageningen, the Netherlands) video tracking system recorded the total time spent in all three zones (open arms, closed arms, and decision zone), as well as the number of entries into the decision zone. The percent time spent in each set of arms was used as a metric of anxiety-like behavior. More time spent in the closed arms is indicative of increased anxiety, and more time spent in the opens arms is indicative of decreased anxiety. The maze was cleaned between trials with Peroxigard Wipes.

### Morris Water Maze

Hippocampal-dependent spatial learning and memory were assessed using the Morris water maze paradigm (MWM) (39, 40) at 1-month post-impact, as previously described (27). Spatial learning and memory are known to be disrupted in clinical TBI, with the Morris Water Maze being used to investigate such deficits in both humans and rodents (35, 41–43). Here, performance on the MWM will serve as a negative control to ensure our impacts are not generating clinical symptomology. MWM testing was conducted in a white pool (83 cm diameter, 60 cm deep) that was filled with water to a 29 cm depth. The water temperature was maintained at 24°C, and a target platform (a round, white, plastic platform 10 cm in diameter) was positioned approximately 1 cm below the water surface. Several highly visible maze cues were located on the wall of the pool. During the hidden and visible platform trials, mice were randomized to one of four starting quadrants (N, S, W, E). At the start of each run, mice were placed in the tank facing the wall and given 90 s to find and mount the platform, and remain on it for 5 s. The latency for the animal to mount the platform (latency to platform) was recorded and used as a measure of learning, with a shorter latency indicating learning. Mice that failed to find and mount the platform within the allotted 90 s were guided to the platform by the experimenter and given 10 s to get familiar with its location. Mice were subjected to one trial a day, consisting of two runs per trial, for 4 days. On the fourth day, following the hidden trial, the mouse also completed a probe trial which tested recall of the platform location. For the probe trial, the platform was removed from the tank and the animal was given 60 s to explore the tank. Noldus Ethovision XT 11.5 software (Wageningen, the Netherlands) was used to track the time spent in the quarter of the pool centered over where the platform was previously located (target counter). Techniques using a more strategic region of the pool, similar to the target counter, as opposed to the target quadrant, to analyze performance have been previously reported (44). The time spent in the target counter was used as a measure of spatial memory; mice with deficits in spatial memory spend less time in the target counter. On the fifth day, the animals performed two visible trials followed by a final probe trial. For the visible platform trials, a red reflector was used to mark the top of the platform, making the platform visible, and mice were again given 90 s to find and mount the platform. Latency to the platform on the visible trials was recorded to account for motivational, or other non-learning-based factors, that might affect performance.

### Von Frey Testing

The Von Frey test evaluates increased tactile allodynia (45) and has been utilized in a variety of rodent models of migraine (46). Inclusion of the test here was driven by the hypothesis that while our low-level RHI protocol should not induce gross cognitive (MWM), affective (Open Field, EPM), or motor (Rotarod) deficits, it may induce subtle changes, such as discomfort, which may be detected by the Von Frey test. After habituation to test environment (7.5 cm × 7.5 cm × 15 cm; 40 min) both the day prior to and the day of testing, post-impact mechanical sensitivity was determined with eight von Frey filaments (bending force of 0.02, 0.04, 0.07, 0.16, 0.4, 0.6, 1, and 2 g) applied to the central part of the hind paw. In between stimuli, if a filament elicited a response, the mouse was allowed to return its paw to the floor, and at least 3 s passed before a new filament was applied. The mechanical response threshold was determined as the minimal force filament to which the animals responded (in at least five of the 10 stimulations).

### Composite Score

To capture the broad effect of such subtle changes across several measures, we derived a composite score for behavior outcomes. This approach has several advantages, including diminishing Type 1 error by reducing the number of outcome measures to a more manageable level, and ultimately improving signal detection by being more sensitive to changes in outcomes. Moreover, composite endpoints characteristically have several other advantages, including being more highly correlated with putative biomarkers and being better at predicting disease progression in disease states such as Alzheimer's Disease.

The behavioral composite score was calculated for Cohort 3 using the sham mean and standard error. A standardized z-score for each behavioral outcome was calculated for every mouse. These scores were then combined to calculate an overall behavioral composite score for each mouse. Each behavioral test was given equal weight as to not overinflate a test that had multiple associated outcomes.

### Euthanasia

All mice were euthanized via cardiac perfusion. Prior to the perfusion animals were deeply anesthetized with a cocktail of ketamine (100–120 mg/kg) and xylazine (100 mg/kg) in sterile saline via IP injection. Mice were placed abdomen up on a surgical pad while small safety needles were used to secure the four paws to allow for easy access to the thoracic cavity. A vertical incision was made from the abdomen to the thorax and the chest flap was secured using a clip to allow access to the heart. A needle was inserted into the left ventricle to take blood. A butterfly needle was then placed into the left ventricle and the right atrium was cut, followed by perfusion with phosphate saline buffer (PBS). The brain was removed, the left hemisphere was post-fixed in 4% paraformaldehyde solution (PFA) for histology, and the right hemisphere was dissected and stored in a −80 degree Celsius freezer for protein analysis. The blood was spun down (5,000 RPM for 5 min), the serum was collected, and stored in a −80 degree Celsius freezer for biomarker analysis.

### Immunohistochemistry

Following post-fixation, brains were moved to 30% sucrose, a cryoprotectant. They were then flash frozen and sliced at 50 μm using a Leica CM1950 cyrostat (Leica Biosystems, Buffalo Grove, IL). The sections were stored in PBS with 0.01% sodium azide until use. Several frontal sections, as well as several caudal sections featuring the hippocampus, were selected and stained using anti-Iba1 Rabbit (1:200, WAKO) and anti-GFAP Rabbit (1:600, Cell Signaling). Briefly, sections were treated with hydrogen peroxide and incubated in blocking solution with 3% normal goat serum prior to being incubated in primary antibody overnight. The following day, sections were washed and incubated sequentially with the appropriate secondary antibody, Vectastain Elite ABC Kit (Vector, Burlingame, CA), and diaminobenzadine (DAB), and mounted with Permount (Thermo Fisher Scientific, Waltham, MA). Slides were imaged at 40x using the MoticEasyScan (Motic Microscopes, San Antonio, TX).

### Fluoro-Jade C Staining

To evaluate potential neuronal death after impact, Fluoro-Jade C staining was conducted using the Ready-to-Dilute Staining Kit according to the manufacturer's protocol (Biosensis, United States, catalog # TR-100). Briefly, tissue sections were mounted and allowed to dry. The slides were then rehydrated in an ethanol and sodium hydroxide solution, followed by 70% ethanol, and finally distilled water. Subsequently, the slides incubated in a solution of 10% potassium permanganate and were rinsed in distilled water. Then, in low light, the slides were soaked in a mixture of 9% Fluoro-Jade C solution and 9% DAPI, before undergoing three separate rinses in distilled water. Slides were coverslipped with DPX mounting media (Sigma-Aldrich, Burlington, MA, catalog #06522). Images were captured at 10x using a Nikon Eclipse Ti microscope (Melville, New York).

### Quantitative Analysis of Molecular Expression in Peripheral Serum and Brain Tissue Using Single Molecule Arrays (Simoa)

Levels of glial fibrillary acidic protein (GFAP), neurofilament light chain (NF-L), Interleukin-1β (IL-1β), and Interleukin-6 (IL-6) were quantified in the serum and cortical tissue lysates using single molecule array technology (Simoa, Quanterix, Billerica, MA). GFAP, a brain-specific marker of astrocytes, was selected due to its identification in the serum following mild-to-moderate TBI (47–49). Similarly, NF-L, a marker of axonal integrity, has been found to change in response to both TBI (50), as well as sub-concussive impacts (8–10). While the diagnostic value of serum NF-L in sub-concussion has more heavily been explored, both NF-L and GFAP require further investigation as potential markers, especially in regard to the associated neural correlates. Although NF-L and GFAP were both attractive target biomarkers because of their establishment in the TBI literature, it was unclear if a single cluster of low-level RHIs would cause sufficient damage to generate changes in either measure. For this reason, we included the cytokines IL-1β and IL-6 to capture subtler changes in pathology. Both are implicated in the inflammatory cascade following TBI in humans and rodents (51), including some evidence that they may influence outcomes.

To prepare the brain lysates, cortical tissue was lysed in RIPA buffer (Cell Signaling Technology, Danvers, MA), 50 mM Tris-HCl, pH 7.4, 150 mM sodium chloride, 1 mM ethylenediaminetetraacetic acid, 1% NP-40, 1% Sodium Deoxycholic acid, 0.1% sodium dodecylsulfate, 1 mM phenylmethylsulfonyl fluoride, protease inhibitor cocktail, and phosphatase inhibitor cocktail (Santa Cruz Biotechnology, Dallas, TX). Prior to the assay for NF-L and GFAP, the tissue was diluted with double-distilled water to reach a final total protein concentration of 4 ug/ml. For the IL-1β and IL-6 assays, two sets of tissue samples were diluted to a final total protein concentration of 600 ug/ml.

The Simoa Neurology 2-Plex B Kit (NF-L, GFAP) was run according to the manufacturer's guidelines. Briefly, samples were thawed, vortexed, and centrifuged at 10,000 G for 5 min. For cortical lysate samples, the supernatant was further diluted with double-distilled water, and for serum samples, the supernatant was directly loaded into the 96-well plate. All samples were run at a four-times dilution, along with eight calibrators run neat and two controls also run at a four-times dilution. The data were validated via a calibration curve with R^2^ ≥ 0.99. For NF-L, the assay had a dynamic range of 0–2,000 pg/ml with a functional lower limit of quantification (LLOQ) of 0.200 pg/ml. For GFAP, the dynamic range was 0–40,000 pg/ml with a functional LLOQ of 4.15 pg/ml.

Similarly, the Simoa Mouse IL-1b Discovery Kit was run according to the manufacturer's guidelines, with the addition of reagent preparation. For these steps, the beads were washed and re-suspended, the detector and SBG reagents were diluted, and the sample diluent was transferred, as outlined in the kit instructions. The assay had a dynamic range of 0–120 pg/ml with a functional lower limit of quantification (LLOQ) of 0.021 pg/ml.

The Simoa Mouse IL-6 Discovery Kit was similarly run according to manufacturer's guidelines, including reagent preparation. The assay had a dynamic range of 0–700 pg/ml with a functional lower limit of quantification (LLOQ) of 0.120 pg/ml.

For all samples that fell under the LLOQ but above the limit of detection (LOD), which means that there was detectable protein but the exact value could not be determined, they were assigned a value midway between the LOD and LLOQ for the specific assay (0.1325 pg/ml for NF-L, 2.3125 pg/ml for GFAP, 0.0125 pg/ml for Il-1b, 0.0775 pg/ml for IL-6) to allow for inclusion of the sample in the statistical analysis. Similarly, for samples where the value fell below the LOD, indicating that there was no detectable analyte, the sample was assigned a value of 0 pg/ml.

### Immunohistochemistry Analysis

For the Iba1 and GFAP staining, images were analyzed using QuPath (52, 53). Cell detectors were created to count positive, stained cells. Briefly, for each stain, a cell detector was generated taking into account stain darkness and cell size. Performance of the cell detector was confirmed via analysis of a detection map. The cell count was performed in the cortex, using the Allen Mouse Brain Atlas (54) as a reference (55).

### Statistical Analysis

Sample size estimates were made based on our prior studies and pilot data, which have indicated that an n of 4 to 8 is generally sufficient for histological and behavioral outcomes (25, 27).

Data are presented as mean ± standard error of the mean. All histological and biomarker measures reflect fold change values normalized using the sham group. For behavioral outcomes, continuous variables were compared by looking at the effects of impact (2 hits/4 h vs. 4 hits/4 h vs. 8 hits/4 h vs. sham) using analysis of variance (ANOVA), or repeated measures ANOVA where appropriate, and Tukey *post-hoc* tests. For histology and biomarkers, all analyses were performed within a single time point using linear regression with robust standard errors to handle non-normally distributed, non-homogenous residuals. All comparisons were performed using sham as the referent group. A cut off of *p* < 0.05 was used for statistical significance. All statistical analysis was performed in R (56). All graphs were made using GraphPad Prism 8 (San Diego, CA) and all figures were made using Affinity Publisher (Serif, West Bridgford, UK).

## Results

### Behavioral Results

#### Latency to Right

**Figure 3:**
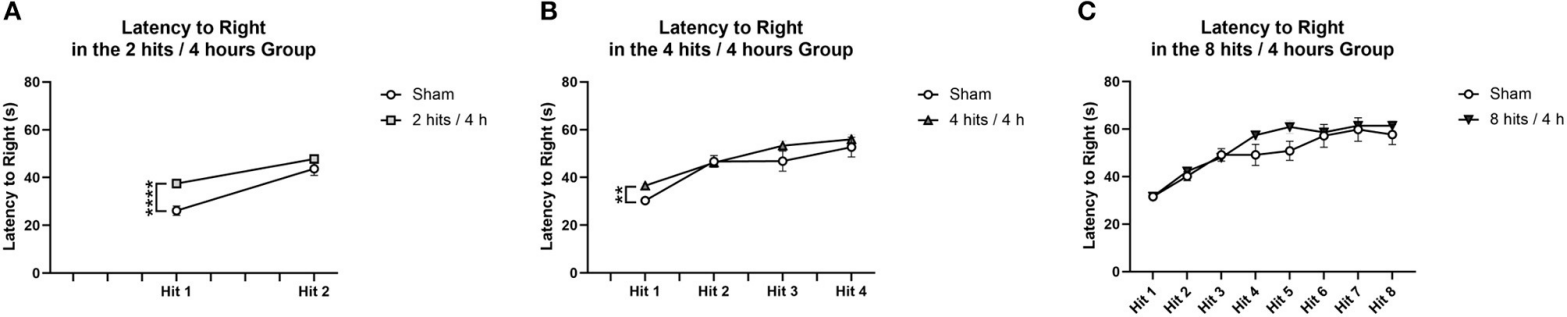
Latency to right was measured as the time from when the animal was removed from isoflurane until the time when the animal was sternal and walking around. Each animal's latency to right was measured, impact condition averages were calculated, and then compared to the sham animals that received the same number of isoflurane exposures. **(A)** The animals that received 2 hits/4 h were compared to sham that received 2 isoflurane exposures. There was a significant difference in latency to right between impact and sham following the first impact (T(25) = −4.84, *p* = 0.00061****), but no difference between groups following the second impact (T(25) = −1.36, *p* = 0.22). **(B)** The animals that received 4 hits/4 h were compared to sham that received 4 isoflurane exposures. There was a significant difference in latency to right between the impact conditions following the first impact (T(26) = 11.34, *p* = 0.0036**). There were no differences between impact groups on loss for consciousness following impact 2 (T(26) = 0.21, *p* = 0.84), impact 3 (T(26) = −1.38, *p* = 0.20), or impact 4 (T(26) = −0.71, *p* = 0.49). **(C)** The animals that received 8 hits/4 h were compared to the sham that received 8 isoflurane exposures. There were no significant differences in latency to right due to impact (Impact 1: T(26) = −0.013, *p* = 0.99; Impact 2: T(26) = −0.88, *p* = 0.39; Impact 3: T(26) = 0.36, *p* = 0.73; Impact 4: T(26) = – 1.72, *p* = 0.12; Impact 5: T(26) = −2.06, *p* = 0.061, Impact 6: T(26) = −0.28, *p* = 0.79, Impact 7: T(26) = −0.29, *p* = 0.78; Impact 8: T(26) = −0.78, *p* = 0.46) for the 8 hits animals.

Comparisons were performed between impact and sham animals within each condition. For the latency to right in the 2 hits/4 h group and 4 hits/4 h group, there was a difference following the first impact, but not after the subsequent impacts (Figures 3A,B). For the 8 hits/4 h group, there were no significant differences in latency to right due to impact (Figure 3C).

#### Cohorts 1 & 2: Locomotor Activity

**Figure 4:**
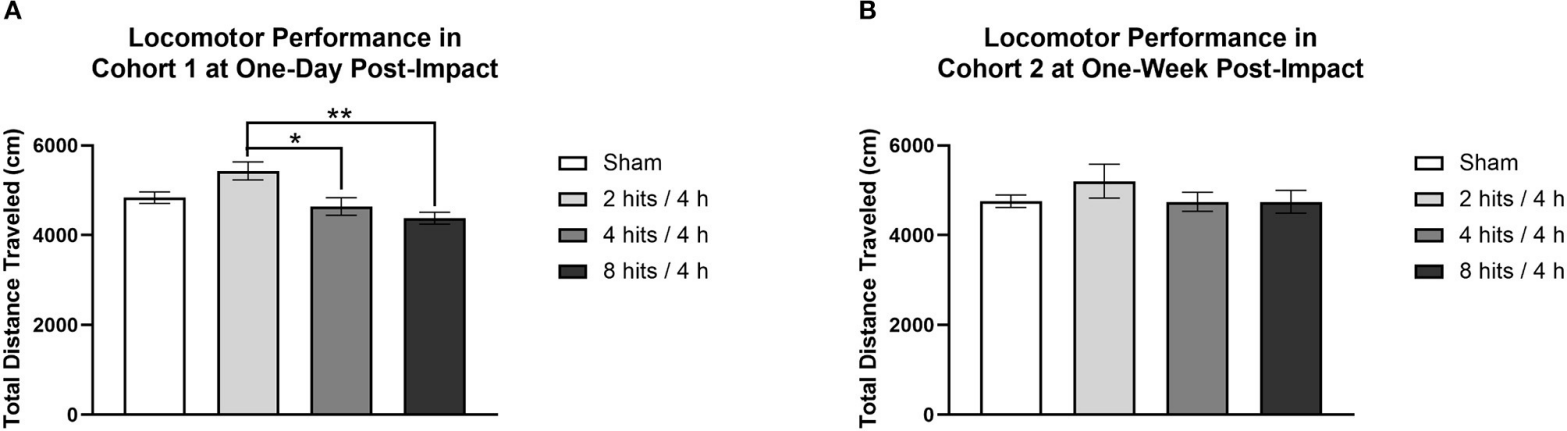
Locomotor activity was measured for each cohort of animals as the total distance traveled (cm) over a 10-min period. **(A)** Cohort 1 underwent locomotor testing at 1-day post-impact. There was a main effect of impact condition for Cohort 1 (F(3, 20) = 7.04, *p* = 0.0020**), with *post-hoc* tests revealing that the 2 hits group (5432.17 ± 200.13) had significantly higher locomotor activity than the 4 hits (4539.33 ± 199.16; *p* = 0.017*) and 8 hits animals (4377.50 ± 135.22; *p* = 0.0014**) (sham: 48.36 ± 127.41; other ps > 0.09). **(B)** Cohort 2 underwent locomotor testing at 1-week post-impact. There was no main effect of impact condition for Cohort 2 (F(3, 20) = 0.77, *p* = 0.53).

Locomotor activity was evaluated at 1-day post-impact in Cohort 1, which revealed a main effect of impact condition. The 2 hits group had significantly higher locomotor activity than the 4 hits and 8 hits (Figure 4A). Locomotor activity was also evaluated at 1-week in Cohort 2. This revealed no effect of the impact protocol at this time (Figure 4B).

#### Cohort 3: 1-Month Behavioral Battery

**Figure 5:**
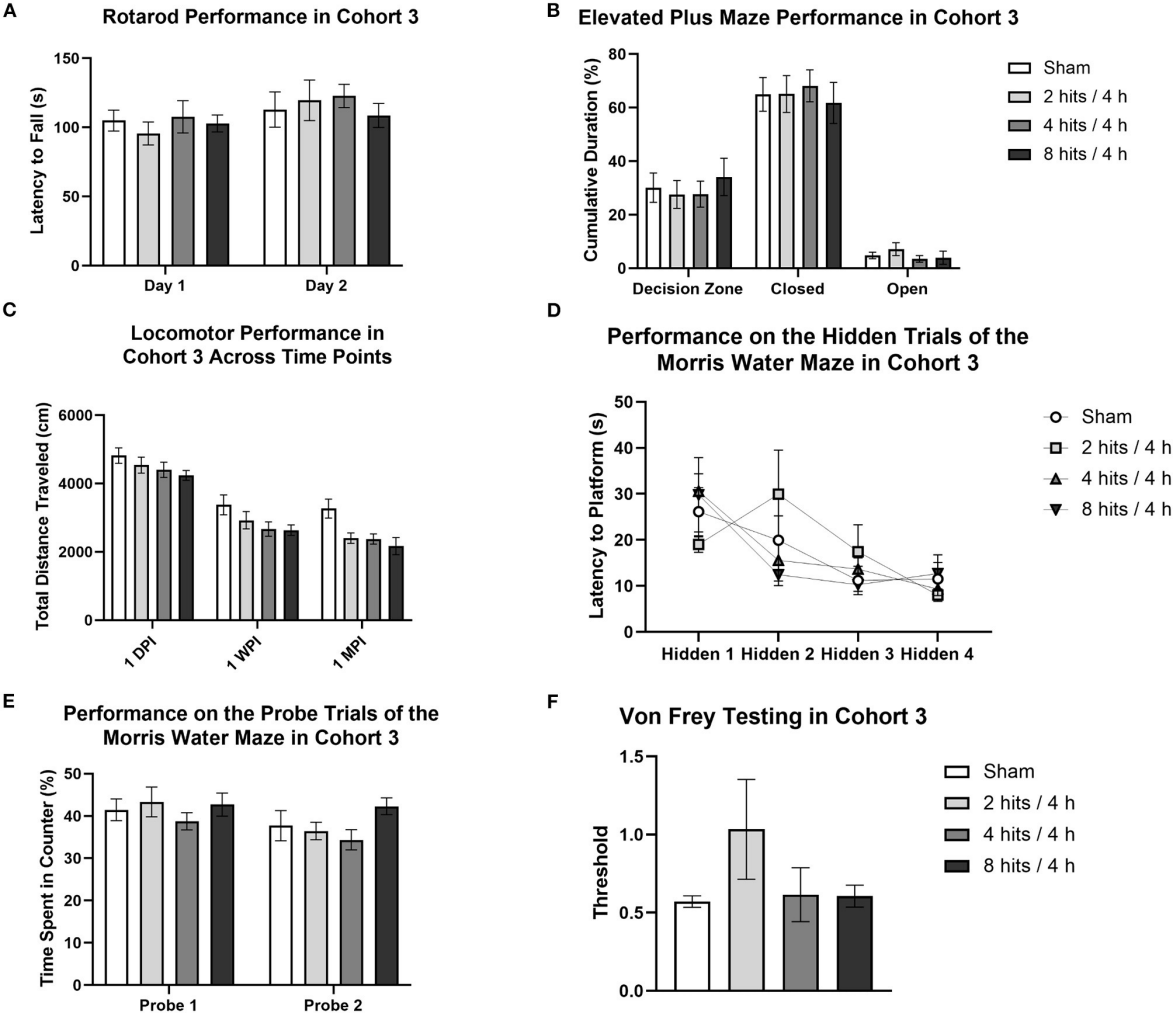
Behavioral testing for Cohort 3 began 2-weeks post-impact and lasted for 2 weeks. **(A)** For rotarod, the latency to fall off of the rotating rod was used as a measure of motor function. Latency to fall was not significantly different between impact conditions (Day 1: F(3,28) = 0.35, *p* = 0.79; Day 2: F(3,28) = 0.31, *p* = 0.82). **(B)** For the Elevated Plus maze, cumulative duration spent in the decision zone, closed arms, and open arms was measured to quantify anxiety-like behavior. Analysis of time spent in the different regions of the maze revealed no differences between groups (Open Arms: F(3, 28) = 0.71, *p* =0.055; Closed Arms: F(3, 28) = 0.15, *p* = 0.93; Decision Zone: F(3, 28) = 0.29, *p* = 0.83). **(C)** Locomotor activity, or voluntary movement, was calculated as total distance traveled. A repeated-measures ANOVA revealed a main effect of impact condition (F(3, 28) = 3.85, *p* = 0.020*) and of testing time point (F(2, 56) = 182.60, *p* < 0.0001***), but no interaction between the two factors (F(6, 56) = 0.85, *p* = 0.54). For the effect of impact condition, Tukey *post-hoc* tests revealed a significant lower distance traveled in the 8 hits/4 h (*p* = 0.018*, 3013.08 ± 212.00) group as compared to sham (3820.13 ± 208.48) and a similar trend for the 4 hits/4 h group as well (*p* = 0.061, 3147.88 ± 215.47 cm) (2 hits/4 h: 3288.00 ± 224.68). All other impact condition comparisons were non-significant (ps > 0.18). For the effect of time point, Tukey *post-hoc* tests revealed significant differences between testing 1-day post-impact, 1-week post-impact, and 1-month post-impact (ps <0.006**). At one-day post-impact, the mean distance traveled in the task (4498.81 ± 107.16 cm) was greater than at 1-week post-impact (2900.72 ± 122.94 cm), and both were greater than the mean distance traveled at 1-month (2552.28 ± 126.82 cm). Additionally, in the Open Field, there were no differences between conditions in time spent in the periphery (F(3,28) = 1.47, *p* = 0.24) or in the center zone (F(3,28) = 1.47, *p* = 0.24). **(D)** The Morris Water Maze was used as a measure of learning and memory. Four hidden trials were completed, each consisting of the averaged performance of two sub-trials. The latency to the platform was recorded for each trial. Repeated-measures ANOVA analysis of the hidden trials revealed no effect of condition (F(3, 28) = 0.16, *p* = 0.92) or interaction between condition and trial number (F(9,84) = 1.50, *p* = 0.16), but did reveal a main effect of trial number (F(3, 84) = 9.43, *p* < 0.0001***). Post-hoc tests indicated a significantly shorter latency to the platform in trials 3 (VS trial 1: *p* = 0.0007***) and 4 (VS trial 1: *p* < 0.0001***, VS trial 2 *p* = 0.033*). **(E)** Probe trials were also run for the Morris Water Maze, as a measure of recall. Performance on the probe trials was measured as the percent time spent in the quarter of the pool centered over the platform, the target counter. Time spent in the target counter during the probe trials was unaffected by impact condition (Probe 1: F(3,28) = 0.54, *p* = 0.66; Probe 2: F (3, 28) = 1.69, *p* = 0.19). Additionally, latency to the platform on the visible trials did not differ as a result of condition (Visible 1: F(3,28) = 1.00, *p* = 0.41; Visible 2: F(3,28) = 0.43, *p* = 0.73). **(F)** Von Frey testing was run as a metric of tactile allodynia and was measured as the threshold at which the animal reliably removed its paw from the stimulus. Response threshold was unaffected by impact condition (F(3, 28) = 1.39, *p* = 0.27).

Our behavioral battery at 1 month (Cohort 3) revealed no motor deficits (Rotarod, Figure 5A), changes in anxiety (Elevated Plus Maze, Figure 5B; Open Field Test), spatial learning and memory deficits (Morris Water Maze, Figures 5D,E), or modulated pain sensitivity (Von Frey Test, Figure 5F).

Locomotor activity was evaluated in the 1-month cohort across time (1-day, 1-week, and 1-month post-impact), which revealed a main effect of impact condition and of testing time point, but no interaction between the two factors (Figure 5C). For the effect of impact condition, the RHI animals that received 4 hits/4 h and 8 hits/4 h traveled a significantly shorter distance than did the sham or 2 hits/4 h. This suggests that the impact protocol, when increased to 4 hits in 4 h and above (8 hits in 4 h), generates a decrease in locomotor activity that persists through the 1-month post-impact time point. For the effect of time point, distance traveled across all groups decreased from 1-day to 1-week to 1-month post-impact. This overall change in distance traveled as a result of time point is likely associated with repeated testing. It appears that when the task is novel, all animals exhibit greater locomotion (likely driven by exploration), but as the novelty decreases with repeated exposures, so do activity levels.

**Figure 6:**
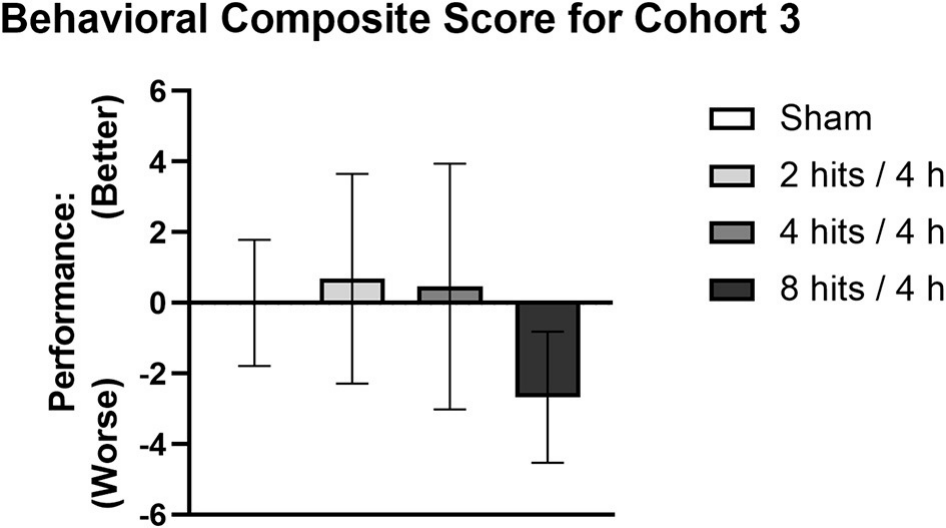
A composite score was generated for Cohort 3 as a cumulative measure of behavioral performance at 1-month post-impact. Each mouse's performance was normalized using sham mean and standard error for the given metric, the new values were then weighted and totaled to generate the composite score. A higher behavioral composite score represents better performance whereas a lower score represents worse performance.

Composite scores for behavioral outcomes were generated at the 1-month time point to capture how subtle differences in behavior across different domains may be additive (Figure 6). This is particularly relevant as, in the acute time frame, sub-concussive blows are not clinically associated with large cognitive or affective changes, but may be able to be represented by small changes across different metrics that rodent behavioral tests may be less equipped to detect. The 8 hits/4 h group showed a decrement on the behavioral composite score compared with the sham, 2 hits/4 h, and 4 hits/4 h, although this difference did not reach significance.

### Histological Results

#### Serum Biomarkers

**Figure 7:**
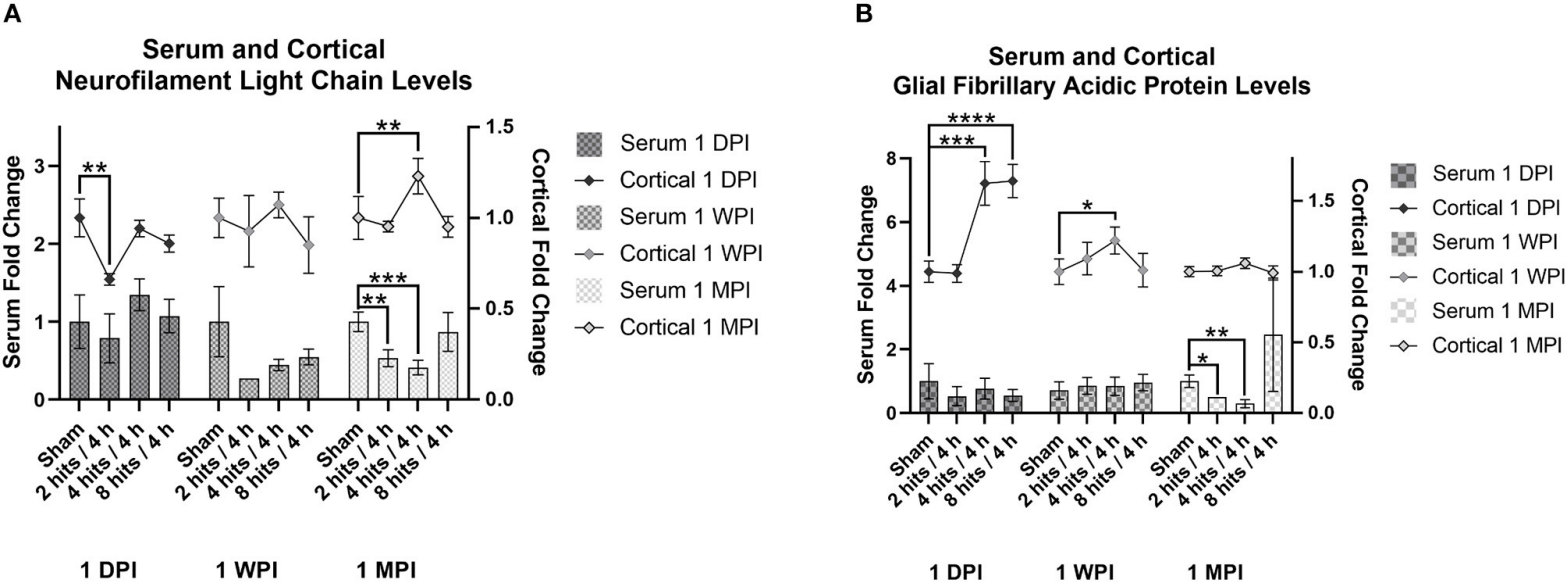
The concentration of Neurofilament Light Chain (NF-L) and glial fibrillary acidic protein (GFAP) in the serum and cortical tissue was measured for all impact groups at each time point. The serum levels are represented via the bar graph with the units on the left Y-axis, and the cortical tissue levels are represented via the line graph with the units on the right Y-axis. **(A)** NF-L levels for both the serum and the cortical tissue are presented as fold change. For serum NF-L, at both 1-day (Cohort 1) and 1-week post-impact (Cohort 2), none of the impact groups were significantly different from sham (Cohort 1: ps > 0.35, Cohort 2: ps > 0.09). At 1-month (Cohort 3), there are significant differences between sham and the 2 hits (*p* = 0.0057**) and 4 hits (*p* = 0.0005***) groups. For cortical NF-L, Cohort 1 (1-day post-impact) showed decreased cortical NF-L in the 2 hits group (*p* = 0.0048**), but no changes in either the 4 hits (*p* = 0.58) or 8 hits (*p* = 0.26) groups. At 1-week post-impact (Cohort 2), there were no differences between impact groups (ps > 0.21), and at 1-month post-impact (Cohort 3), cortical NF-L was elevated in the 4 hits animals (*p* = 0.0082**), but in neither the 2 hits (*p* = 0.65) nor the 8 hits groups (*p* = 0.73). **(B)** GFAP levels for both the serum and the cortical tissue are presented as fold change. For serum GFAP, levels were not different between impact conditions at 1-day (Cohort 1, ps > 0.34) or 1-week post-impact (Cohort 2, ps > 0.58). At 1-month post-impact (Cohort 3), serum GFAP was significantly decreased in the 2 hits (*p* = 0.024*) and 4 hits/4 h groups (*p* = 0.0092**), but the 8 hits group remained at sham levels (*p* = 0.37). For cortical GFAP, expression was significantly elevated in the 4 hits (*p* = 0.0008***) and 8 hits groups (p > 0.0001****), but not the 2 hits (*p* = 0.93) at 1-day post-impact (Cohort 1). This increase returned to sham levels at 1 week (Cohort 2) (ps > 0.16), although there was a small increase in the 4 hits group at 1-month (Cohort 3) (*p* = 0.047*).

Serum NF-L levels were evaluated within each time point for the effects of impact condition (Figure 7A). At both 1-day (Cohort 1) and 1-week post-impact (Cohort 2), none of the impact groups were significantly different from sham in terms of serum NF-L. Visually, it does appear like there may be a subtle trend toward an increase in serum NF-L in response to impact immediately following, but at 1-week, levels of serum NF-L look depressed. At 1-month (Cohort 3), there are significant differences between sham and the 2 hits and 4 hits groups, both of which exhibit decreases in serum NF-L in comparison to sham. The 8 hits/4 h group is comparable to sham at 1 month.

Serum GFAP levels were not different between impact conditions at 1-day or 1-week post-impact (Figure 7B). At 1-month post-impact (Cohort 3), serum GFAP was significantly decreased in the 2 hits and 4 hits/4 h groups, but the 8 hits group remained at sham levels. Although not statistically significant, likely due to the large variability, the 8 hits/4 h group does have a notably higher mean than the sham group at 1 month.

**Figure 8:**
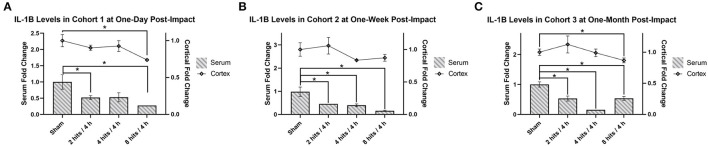
The concentration of Interleukin-1β (IL-1β) in the serum and cortical tissue was measured for all impact groups at each time point. The serum levels are represented via the bar graph with the units on the left Y-axis, and the cortical tissue levels are represented via the line graph with the units on the right Y-axis. IL-1β levels for both the serum and the cortical tissue are presented as fold change. **(A)** Cohort 1 was measured at one-day post-impact. For serum IL-1β, there was significantly lower expression in the 2 hits and 8 hits groups (ps <0.05*), with the 4 hits/4 h group approaching significance (*p* = 0.063). For cortical IL-1β, there was a significant decrease in the 8 hits group at 1-day post-impact (Cohort 1) (*p* = 0.0028**). **(B)** Cohort 2 was measured at 1-week post-impact. For serum IL-1β, there was significantly lower expression across all impact groups (ps <0.05*). For cortical IL-1β, there were no differences between groups (ps > 0.1). **(C)** Cohort 3 was measured at 1-month post-impact. For serum IL-1β, there was significantly lower expression across all impact groups (ps <0.05*). For cortical IL-1β, the significant decrease observed in the 8 hits group returned (*p* = 0.035*) (all other ps > 0.35).

Analysis of serum IL-1β revealed significantly lower expression across all time points (Cohort 1–3) and across all impact groups, with the exception of the 4 hits/4 h group at 1 day after impacts, which was approaching a significant decrease as well (Figure 8).

**Figure 9:**
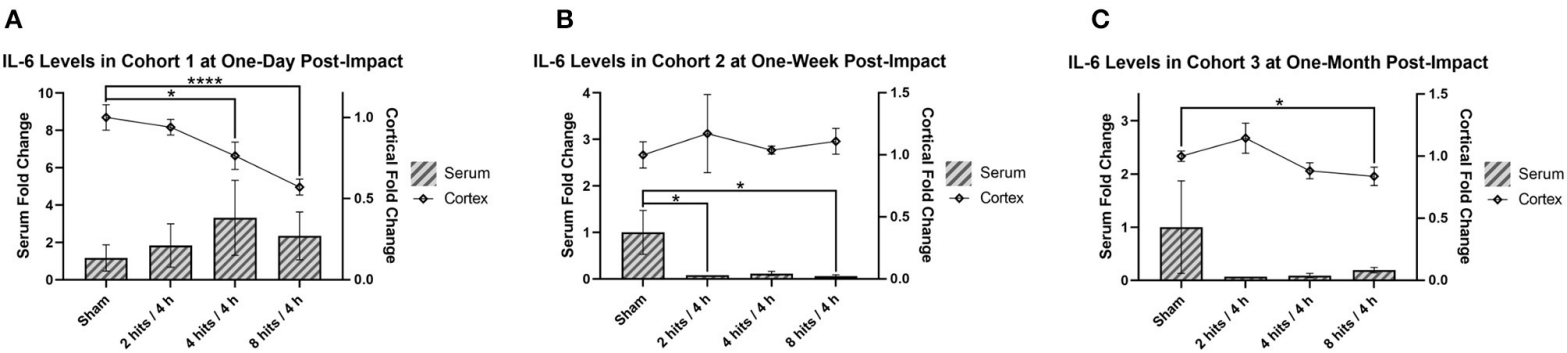
The concentration of interleukin-6 (IL-6) in the serum and cortical tissue was measured for all impact groups at each time point. The serum levels are represented via the bar graph with the units on the left Y-axis, and the cortical tissue levels are represented via the line graph with the units on the right Y-axis. IL-6 levels for both the serum and the cortical tissue are presented as fold change. **(A)** Cohort 1 was measured at 1-day post-impact. For serum IL-6, there were no significant differences between groups (ps > 0.28). For cortical IL-6, there was a significant decrease in the 4 hits (*p* = 0.036*) and 8 hits groups (*p* < 0.0001****) compared to sham (2 hits: *p* = 0.48). **(B)** Cohort 2 was measured at 1-week post-impact. For serum IL-6, both the 2 hits (*p* = 0.045*) and 8 hits (*p* = 0.041*) animals exhibited significantly decreased serum IL-6 compared with sham, while the 4 hits group approached significance in the same direction (*p* = 0.054). For cortical IL-6, there were no differences between groups (ps > 0.11). **(C)** Cohort 3 was measured at 1-month post-impact. For serum IL-6, there were no significant differences between groups (ps > 0.25). For cortical IL-6, the significant decrease in the 8 hits group was evident again (*p* = 0.050*).

Serum IL-6 was not significantly different as a result of impact at 1 day, but did trend toward an increase in response to impact (Figure 9A). At 1-week post-impact, both the 2 hits and 8 hits animals exhibited significantly decreased serum IL-6 compared with sham, while the 4 hits group approached significance in the same direction (Figure 9B). While non-significant, at 1 month (Cohort 3), the trends toward decreased IL-6 in the serum of impact animals persisted (Figure 9C).

#### Cortical Analytes

Analysis of cortical NF-L was also performed across impact conditions within each time point (Figure 7A). Cohort 1 (1-day post-impact) showed decreased cortical NF-L in the 2 hits group, but no changes in either the 4 hits or 8 hits groups. At 1-week post-impact (Cohort 2), there were no differences between impact groups, and at 1-month post-impact (Cohort 3), cortical NF-L was elevated in the 4 hits animals, but in neither the 2 hits nor 8 hits groups.

Cortical GFAP expression was significantly elevated in the 4 hits and 8 hits groups, but not the 2 hits at 1-day post-impact (Cohort 1) (Figure 7B). This increase returned to sham levels at 1 week (Cohort 2), although there was a small increase in the 4 hits group at 1-month (Cohort 3).

IL-1β in the cortex was significantly decreased in the 8 hits group at 1-day post-impact (Cohort 1) (Figure 8A), and while it did not reach significance, similar trends were seen in the 2 hits and 4 hits/4 h groups at this time. At 1 week (Cohort 2), all groups were comparable to sham (Figure 8B), but at 1-month post-impact (Cohort 3), the significant deficit observed in the 8 hits group returned, although all other impact groups remained non-significant (Figure 8C).

Cortical IL-6 was significantly decreased in the 4 hits and 8 hits groups compared to sham at 1-day post-impact (Figure 9A). Levels had returned to sham at 1 week (Cohort 2) (Figure 9B), but at 1-month post-impact (Cohort 3) the significant decrease in cortical IL-6 in the 8 hits group was evident again (Figure 9C).

#### Immunohistochemistry

**Figure 10:**
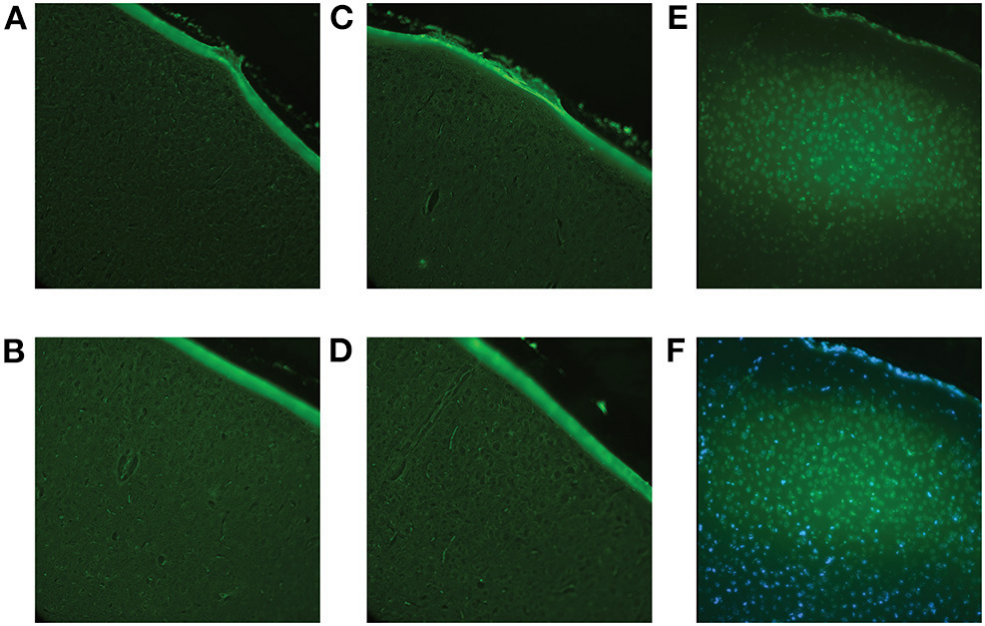
Fluoro-Jade C staining was performed to confirm that the impact procedure did not induce immediate cell death. Using the brains of the animals sacrificed at 1-day post-impact (Cohort 1), all brain regions were examined to ensure accuracy. **(A)** Representative image of the sham cortex with no visible cell death. **(B)** Representative image of the cortex (location of impact) of a 2 hits/4 h animal with no visible cell death. **(C)** Representative image of the cortex of a 4 hits/4 h animal with no visible cell death. **(D)** Representative image of the cortex of an 8 hits/4 h animal with no visible cell death. **(E)** A positive control, a controlled cortical impact brain, where there is known cell death, was included for reference. The controlled cortical impact brain shows definite positive staining. **(F)** Overlay of Fluoro-Jade and DAPI staining of the controlled cortical impact brain to confirm that the positive signal was, in fact, representative of cell death.

Fluoro-Jade staining confirmed no cell death in response to low-level RHI at all impact frequencies (Figure 10).

**Figure 11:**
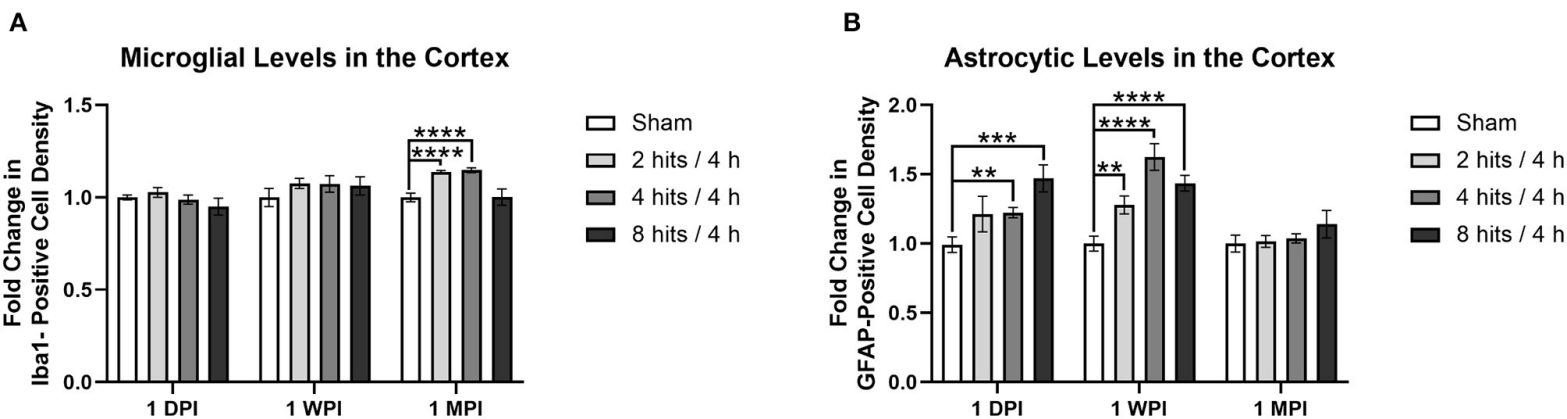
Immunohistochemistry was performed with an n of 4 per impact group across each cohort. A cell count was performed in the cortex and the results are presented as fold change calculated using the sham average. **(A)** Average microglial density was measured via Iba1 positivity. There were no changes in any of the impact groups at 1 day (Cohort 1, ps > 0.24) or 1 week (Cohort 2, ps > 0.14), but at 1 month, there were significant increases in the 2 hits (*p* < 0.0001****) and 4 hits groups (*p* < 0.0001****), but not the 8 hits/4 h group (*p* = 0.97). **(B)** Average astrocytic density was measured via GFAP positivity. At one-day post-impact, there was a significant increase in the 4 hits (*p* = 0.0019**) and 8 hits (*p* = 0.0003***) groups, and a trend toward an increase in the 2 hits/4 h group (*p* = 0.095). At 1-week post-impact (Cohort 2), there was a significant increase in astrocytes in all impact groups in comparison to sham (2 hits: *p* = 0.0026**, 4 hits: *p* < 0.0001****, 8 hits: *p* < 0.0001****). At 1-month, levels were comparable to sham (Cohort 3, ps > 0.19).

Analysis of the fold change in microglia cell count (via Iba1 staining) in the cortex demonstrated no changes in any of the impact groups at 1 day (Cohort 1) or 1 week (Cohort 2) (Figure 11A). At 1 month (Cohort 3), changes began to emerge in which significant increases in microglia were observed in the 2 hits and 4 hits groups, but, interestingly, the 8 hits/4 h animals remained at sham levels.

Fold change in astrocyte cell count (via GFAP staining) in the cortex revealed a significant increase in the 4 hits and 8 hits groups, and a trend toward an increase in the 2 hits/4 h group at 1 day (Cohort 1) (Figure 11B). At 1-week post-impact (Cohort 2), there was a significant increase in astrocytes in all impact groups in comparison to sham, but this dissipated at 1 month (Cohort 3).

#### Fold Change Heatmaps

**Figure 12:**
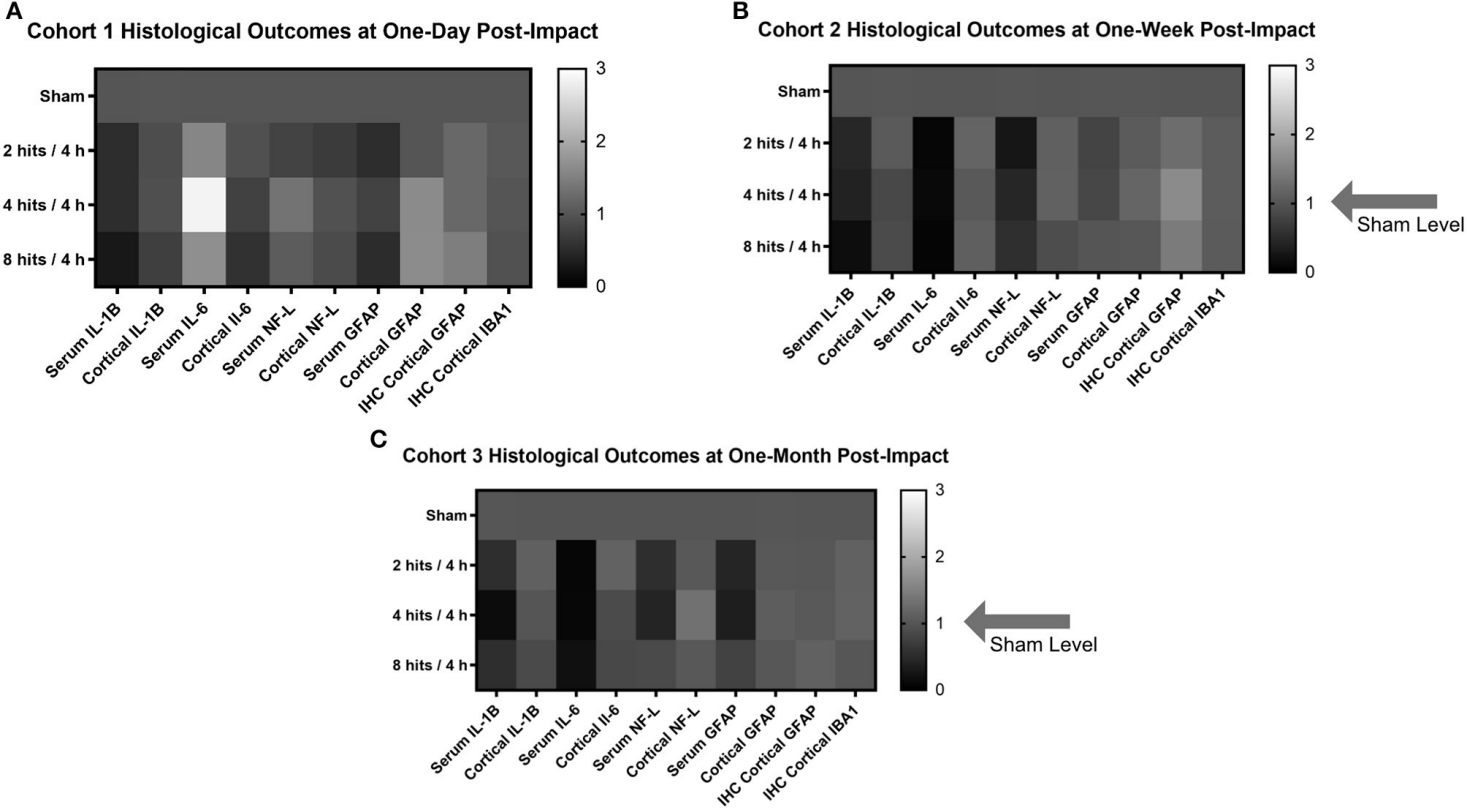
Heat maps were generated to look at the trends in the biomarker and histological outcomes in each impact group for each cohort. Values were calculated by computing the fold change in each cohort compared to the sham average at the comparable time point for each outcome measure. **(A)** Histological heat map for Cohort 1 at one-day post-impact. **(B)** Histological heat map for Cohort 2 at one-week post-impact. **(C)** Histological heat map for Cohort 3 at one-month post-impact.

In order to create a visual and summative representation of all molecular assays (blood and tissue), we created a heatmap of the molecular outcomes for each cohort and time point (Figure 12).

## Discussion

Here, we have reported the development of a new model of low-level RHIs, and evaluated both acute and sub-acute behavioral, biomarker, and histological outcomes in response to increased impact frequency within a single session. By including a broad array of behavioral outcomes, we offer a comprehensive functional assessment following low-level RHIs. Our characterization of the biomarker and histological responses offer unique data, which implicate inflammatory and immune processes in the resulting pathology.

While we saw subtle behavioral differences in individual and composite behavioral assays (i.e. changes in locomotion at 1 month, deficits on composite performance in the 8 hits group), it is notable that one cluster of RHIs, regardless of frequency, induced histological changes in the brain. Changes in astrocytes, microglia, and cytokines in the cortex all revealed a dose-dependent response to impact load, with some effects enduring at the sub-acute time point. Further, these changes were observed in previously “naïve” animals, that had no history of either sub-concussive or concussive RHI exposure, which demonstrates that a single cluster of low-level impacts alone, in the absence of pre-existing microstructural damage, is sufficient to produce these changes. These low-level impacts may have a greater effect on brain integrity than initially suspected, with further efforts to characterize brain regions aside from the cortex warranted.

Our cytokine analysis, using Simoa® technology, demonstrated robust decreases in IL-1β and IL-6, which, in light of the existing literature on head trauma, are quite paradoxical. Across all impact groups, and following a dose-dependent pattern, IL-1β and IL-6 were downregulated in both the serum and the cortex, with the effect enduring up to 1-month post-impact. The only exception being IL-6 levels in the serum at 1-day post-impact, which showed the inverse pattern, but also had fairly large variability, which may affect the appearance of such a trend. While IL-1β is recognized as a pro-inflammatory cytokine (54), IL-6 has dual functions, with acute anti-inflammatory properties and chronic pro-inflammatory effects (57–59). Increases in both cytokines are implicated in the signaling cascade and resultant pathology following TBI (from mild to severe), with some evidence that the inflammation contributes to worse outcomes post-injury (51, 60, 61). Yet here, both cytokines were downregulated, representing what seems to be a blunted inflammatory response. Although it may seem inconsistent that both cytokines decreased concurrently, IL-1β serves as one of the primary inflammatory signals, and is upstream from IL-6 (57), so mechanistically, it is possible that a blunted IL-1β response could inhibit and potentially depress the resulting IL-6 response. Hence, these changes could represent an overall trend toward anti-inflammation, or immune suppression. Interestingly, Hiles-Murison et al., also found suppression of several pro-inflammatory chemokines and cytokines (CXCL1 and MCP-1), although they saw no changes in IL-1β or IL-6 (16). In a clinical study, Huibregtse et al. subjected soccer players to 10 controlled headers and tested plasma levels of pro-inflammatory chemokines (CCL11 and CCL2) and an anti-inflammatory cytokine (IL-10) immediately post-heading, as well as 2 h and 24 h later (62). While CCL2 and IL-10 levels remained consistent after heading, plasma CCL11 levels diverged based on previous head impact exposure. Specifically, those who had fewer years of heading exposure (e.g., 3–5 years) showed a reduction in CCL11, which mirrors our data in naïve animals, whereas those with many years of heading exposure (e.g., 10–15 years) showed an increase in CCL11. Differences in impact paradigm, as well as between human and animal models (mice vs. rats), may explain the divergence in which specific markers changed in response to impact, but the anti-inflammatory profile in response to low-level RHIs remains a common theme.

One limitation of exploring non-specific blood biomarkers is the potential for the influence of soft tissue damage, such as the soft tissue envelope of the cranial vault, that was sustained during head impact. While we cannot rule out any potential changes in serum IL-1β or IL-6 due to soft tissue damage, we believe the changes in serum cytokines are most reflective of changes in the brain. Notably, the patterns in serum IL-1β and IL-6 mirror what was observed in our cortical lysates. Importantly, the biomechanical insult in our model is directly delivered to the head, similar to clinical TBI, in which both impact and acceleration insults are frequently sustained. In this way, we believe the modeling translates well to clinical TBI, where injury to the soft tissues of the head may accompany brain injury. However, RHI exposures are often accompanied by extracranial injuries as well, and it is important to further detail how polytrauma could confound the expression of these nonspecific markers. Attention must be paid in the use of non-brain-specific blood biomarkers to monitor changes in the brain, but such measures may still be informative and relevant.

Microglia in the cortex were increased in the 2 hits and 4 hits groups sub-acutely, but the animals subjected to 8 hits/4 h had levels comparable to sham. Work by Honig et al. showed a decrease in Iba1 labeling in response to their impact model, with further analysis revealing changes in microglial morphology that were indicative of a non-primed state (17). While more in-depth microglial analysis was outside of the scope of this experiment, future work to understand if these changes are a hallmark of low-level head impacts is warranted. Additionally, primed microglia are known to both produce and be responsive to IL-1β and IL-6 (57, 63, 64), so it is possible that there could be a direct link between the observed decreases, although the causality or directionality is not evident here. Together, this data suggests that, in the context of early exposures to low-level RHIs, downregulation of inflammatory pathways may play a role in pathology.

One broad trend in the GFAP and NF-L data, although non-significant, is the appearance of a greater increase in cortical, as well as serum, GFAP and NF-L, in the 4 hits group, as compared to the 8 hits group. While this pattern is subtle, it is ubiquitous across time points and cohorts for these markers, which suggests it may be indicative of a real effect. It is difficult to speculate the biological significance or underlying mechanisms, meaning further work is needed to gain an understanding of these changes. Moreover, these results were not entirely consistent with our initial hypotheses. Based on the clinical concussion and sub-concussion literature (8–10, 47–50), we expected to see a robust response in each measure in both the brain and the blood. GFAP in the cortex (via cortical lysate protein quantification and cortical immunohistochemistry) showed immediate increases in response to impact. Serum GFAP showed a much less clear pattern in response to impact, potentially indicating decreases in serum GFAP in both the 2 hits and 4 hits groups, similar to the pattern observed in serum NF-L. The cortical GFAP data may indicate that the low-level impacts cause an astrocytic response, possibly via glutamate release following impact, but not sufficient damage to lead to significant GFAP release into the periphery.

There were also no clear patterns in cortical NF-L in response to impact, which suggests that there was no gross axonal damage in response to one RHI cluster. The serum NF-L results were more interesting, albeit difficult to explain. Trends suggest very subtle initial increases in NF-L in the serum of 4 hits/4 h and 8 hits/4 h animals at 1 day, which follows the direction of what has been observed in the clinical literature (8–10). Interestingly, at 1-day post-impact, the serum and cortical NF-L patterns match, but the changes only reach significance in the cortical measurements. While further work is necessary to investigate this relationship, our hypothesis is that these patterns reflect true changes, and that due to the variability of the biomarker data and subtly of the effects generated by low-level RHIs, a larger sample size would be necessary to achieve significance in the serum data. More pronounced were the decreases observed in serum NF-L across all impact groups at 1 week, which persisted for the 2 hits and 4 hits groups through 1-month post-impact, while the 8 hits group returned to sham levels. Despite clinical demonstrations, in both football (8) and soccer (9, 10), that one cluster of RHI exposures can lead to an increase in serum NF-L, the enduring effect of our model was a decrease in serum NF-L. While the clinical studies appear potentially contradictory to our current study, it is important to note that the clinical studies evaluated pre- and post-session biomarker changes in athletes with a history of RHI exposure. In contrast, as discussed above, the animals included here were fully naïve at the start of the study, which represents a population that does not directly translate to athletes with prior RHI exposure. Although it is surprising that we saw a decrease in serum NF-L, it is not surprising that our data did not align perfectly with the clinical results. We hypothesize that as RHI cluster exposure increases across time, serum NF-L, and potentially GFAP as well, in the mice would better mirror the reported clinical changes and represent an additive measure of RHI load. These results may reveal that early in the course of low-level RHI exposure (i.e. early in an athlete's career), blood levels of GFAP or NF-L may not accurately reflect impact load and initial pathology. Exploration of how serum NF-L and GFAP change in response to repeated RHI clusters is warranted. By building on this initial characterization of changes following the first RHI exposure, we can better understand how clusters may be additive, as well as the existence of dose-dependent or threshold patterns.

With the neuronal specificity of NF-L expression, it is unlikely that the decrease in serum NF-L represents a change to the brain, but instead may be related to either decreased transport across the blood-brain barrier (BBB) or a systemic clearance mechanism. Currently, it is not known how NF-L crosses the BBB in non-disease states (65), so it is possible that there may be dysfunction in transport, decreasing the amount of NF-L that is escaping into the periphery. As cortical NF-L has such a high concentration, it is possible that this difference in transport may not be picked up by our cortical protein analysis. Alternatively, in response to impact, a peripheral clearance mechanism may become overactive, decreasing the half-life of serum NF-L and effectively decreasing its serum concentration. Determining which of these mechanisms is driving the disparate serum NF-L results, as well as how it either contributes to pathology or confers protection, may inform our understanding of the timeline of NF-L as a serum biomarker.

It must be kept in mind that this impact paradigm attempts to model the sports-related phenomenon of sub-concussion, as best can be done in an animal model. We are limited by the inability to investigate symptoms of concussion, relying only on signs of injury. Attempts at novel ways to interrogate animals' symptoms, which clinically rely mainly on self-report, will be important to improve accuracy of sub-concussion models.

In summary, our low-level repetitive head impact model uses a clinically-relevant impact intensity and frequency to evaluate the effects of a single RHI cluster both acutely and sub-acutely, revealing not only subtle behavioral deficits with increased RHI frequency, but also histopathology and lasting changes in inflammation in response to a single cluster of RHI. Particularly, several key cytokines were decreased in both the cortex and serum in a dose-dependent manner. While unexpected and counterintuitive in light of what we understand about the pathology of mild TBI and concussion, these results provide a basis for further exploration. Understanding how dose and frequency modify the physiology of injury will be essential in monitoring, treatment, and long-term mitigation of the potentially detrimental effects of head trauma.

## Data Availability Statement

The raw data supporting the conclusions of this article will be made available by the authors, without undue reservation.

## Ethics Statement

All experiments were approved by the Boston Children's Hospital Institutional Animal Care And Use Committee and complied with the NIH Guide for the Care and Use of Laboratory Animals.

## Author Contributions

MB, JQ, GC, WM, JB, KK, and RM were responsible for study concept and study design. MB, JQ, JN, and GC were responsible for acquisition of data. MB and RM were responsible for primary statistical analyses. MB, GC, and RM were responsible for drafting the manuscript. RM takes primary responsibility for the paper as a whole. All authors contributed to data analysis and/or interpretation, and substantially to critical revisions related to important intellectual content. All authors have approved of this submission.

## Funding

This work is part of the NFL-LONG study, which is funded by a grant from the National Football League. Part of the work was performed in the Animal Behavioral and Physiology Core at Boston Children's Hospital (CHB IDDRC, 1U54HD090255).

## Conflict of Interest

RM research is funded by grants from the National Institutes of Health, the Department of Defense, and the National Football League. She received research support by Abbott Pharmaceuticals. WM receives royalties from: 1) ABC-Clio publishing for the sale of his books, Kids, Sports, and Concussion: A Guide for Coaches and Parents and Concussions; 2) Springer International for the book Head and Neck Injuries in Young Athlete; and 3) Wolters Kluwer for working as an author for UpToDate. His research is funded, in part, by philanthropic support from the National Hockey League Alumni Association through the Corey C. Griffin Pro-Am Tournament and a grant from the National Football League. The remaining authors declare that the research was conducted in the absence of any commercial or financial relationships that could be construed as a potential conflict of interest.

## Publisher's Note

All claims expressed in this article are solely those of the authors and do not necessarily represent those of their affiliated organizations, or those of the publisher, the editors and the reviewers. Any product that may be evaluated in this article, or claim that may be made by its manufacturer, is not guaranteed or endorsed by the publisher.
